# Evaluation of transverse and sagittal maxillary arch dimensions in growing subjects with Class II Division 1 and Division 2 malocclusion: a retrospective cross-sectional study

**DOI:** 10.3389/fdmed.2026.1842593

**Published:** 2026-07-22

**Authors:** Franceska Vinjolli, Grazia Marinelli, Rahela Frakulla, Paola Bassi, Francesco Inchingolo, Angelo Michele Inchingolo, Andrea Palermo, Alessio Danilo Inchingolo, Gianna Dipalma

**Affiliations:** 1 Department of Medical Science, Catholic University Our Lady of Good Counsel, Tirana, Albania; 2 Department of Interdisciplinary Medicine, University of Bari “Aldo Moro”, Bari, Italy; 3 Private Practice, Tirana, Albania; 4 Department of Biomedical, Surgical and Dental Sciences, Milan University, Milan, Italy; 5 Department of Experimental Medicine, University of Salento, Lecce, Italy

**Keywords:** arch length, Class II malocclusion, digital models, growing patients, maxillary arch, transverse width

## Abstract

**Objective:**

To examine the transverse and sagittal dimensions of the maxillary arch in developing children with Class II Division 1 and Class II Division 2 malocclusions and compare them to those with Class I malocclusion.

**Materials and methods:**

This retrospective observational study included 60 growing subjects, comprising 32 females and 28 males, with a mean age of 10.5 ± 1.4 years. The sample was divided into three groups: Class I, *n* = 20; Class II Division 1, *n* = 20; and Class II Division 2, *n* = 20. Plaster models were digitized into STL files and assessed using MeshLab software. Transverse widths were measured at the dental-crown level and at the palatal gingival-margin level as complementary assessments of maxillary arch morphology. Maxillary arch length was also assessed. Method error was evaluated through repeated measurements on 15 randomly selected models after 4 weeks. Intraclass correlation coefficient analysis showed excellent intra-operator consistency for all measurements.

**Results:**

The transverse measurements taken at the palatal gingival margin were significantly lower in both Class II groups when compared to Class I subjects. At the cusp level, individuals with Class II Division 1 showed no significant differences compared to those with Class I, while Class II Division 2 individuals remained considerably narrower at both the cusp level and the palatal gingival margin level. In individuals with Class II Division 2, the maxillary arch length was significantly less than in other classifications, while Class II Division 1 and Class I showed no major differences in arch length.

**Conclusions:**

In growing patients, Class II Division 2 and Class II Division 1 malocclusion showed smaller maxillary transverse dimensions. Subjects with Class II Division 2 displayed a reduced maxillary arch length in comparison to the other groups.

## 1 Introduction

Several studies in the literature have analyzed the skeletal and dentoalveolar components of Class II malocclusion in order to improve orthodontic diagnosis and treatment planning. Many investigations have demonstrated differences in maxillary arch morphology in subjects with Class II malocclusion compared with individuals with normal occlusion. With the exception of the study conducted by Fröhlich in 1961 (8), subjects with basal Class II malocclusion have been reported to present a transverse constriction of the maxilla, both skeletally and dentoalveolarly, resulting in a narrower and more elongated arch form (1–8).

In the study by Buschang et al. (15), a sample of 386 female subjects aged 17–68 years was analyzed and stratified by age group and occlusal type. The authors showed that palatal height was greater in younger women than in older women, and that subjects with Class II malocclusion had significantly smaller arches than those with Class I relationships (9–15). Moreover, subjects with Class II Division 1 Malocclusion had higher palates and relatively longer and narrower maxillary arches compared with those with Class II Division 2 Malocclusion. Compared with subjects with Class I occlusion, patients with Class II Division 1 Malocclusion show a significantly narrower transverse maxillary arch diameter both in the lateral incisor–canine region and in the interpremolar and intermolar regions (16–20).

A key aspect in defining the treatment strategy for Class II Malocclusion is understanding whether this type of dysgnathia may undergo spontaneous correction during growth and, if not, at what stage of craniofacial development intervention is most appropriate. The longitudinal study by Stahl and colleagues reported noteworthy findings (28). The authors analyzed craniofacial growth changes in untreated subjects with Class II malocclusion and compared them with those observed in subjects with normal occlusion, from the prepubertal period to the postpubertal period. The study showed that, in the absence of treatment or modifications of etiologic factors, Class II malocclusion does not undergo spontaneous self-correction during growth (21–28).

The shape of the maxillary arch is an important diagnostic parameter that can be modified to improve malocclusion in growing patients. Most studies in the literature have investigated adult samples. Therefore, the null hypothesis was that there would be no statistically significant differences in maxillary transverse dimensions or maxillary arch length among subjects with Class I, Class II Division 1, and Class II Division 2 malocclusions (29–35).

## 2 Materials and methods

### 2.1 Sample size

The study included a retrospective convenience sample of 60 growing subjects, with 20 participants in each malocclusion group. The sample was obtained from the institutional archives of the participating centers and included all available eligible pretreatment records within the defined study period. Therefore, the sample size was determined by the number of available cases meeting the inclusion criteria rather than by an *a priori* sample size calculation. This sampling strategy should be considered when interpreting the generalizability of the findings. The sample included 32 females and 28 males, with a mean age of 10.5 ± 1.4 years. The observed age range of the final sample was 7.8–13.0 years.

Because the sample was obtained from records of two participating centers located in different countries, recruitment center and country of origin were considered potential sources of heterogeneity. Due to the limited sample size, adjusted analyses according to recruitment center or country of origin were not performed, and this aspect was considered when interpreting the findings.

### 2.2 Inclusion criteria

Participants were chosen based on the subsequent inclusion criteria:

- Availability of high-quality pretreatment records, including plaster study models and lateral cephalometric radiographs;
- Caucasian growing subjects aged 7–13 years.
- No prior orthodontic treatment, validated via clinical documentation and patient medical history.
- Participants were categorized based on their dental and skeletal relationships.
- The Class I group comprised individuals with Angle Class I molar and canine relationships, ANB angles ranging from 0° to 4°. The Class II Division 1 cohort consisted of individuals with Angle Class II molar and canine relationships, an ANB angle exceeding 4°, an increased overjet (>4 mm).

The Class II Division 2 group comprised subjects with Angle Class II molar and canine relationships and ANB angle >4°, reduced overjet, increased overbite, palatal inclination of the upper central incisors, and labial inclination of the upper lateral incisors. Subjects with normodivergent, hypodivergent, and hyperdivergent facial patterns were represented in each malocclusion group. However, vertical facial type was not used as an inclusion criterion or stratification variable.

The criteria for exclusion encompassed:

- Lack of back teeth (loss of permanent molars and/or significant decay)
- Occurrence of dental agenesis
- Prior orthodontic treatment
- Craniofacial syndromes, craniofacial anomalies, or cleft lip and/or palate.

The study sample was divided into three groups according to malocclusion type: Class I group: 20 participants (13 females; 7 males) Class II Division 1 category (II-1): 20 participants (8 females; 12 males) Class II Division 2 cohort (II-2): 20 participants (11 females; 9 males).

Potential confounding factors, including age, sex, dentition stage, and recruitment center/country of origin, were considered during interpretation of the results. However, because of the limited sample size, subgroup stratification and adjusted analyses were not performed.

### 2.3 Digital model acquisition and measurement protocol

**Figure 1:**
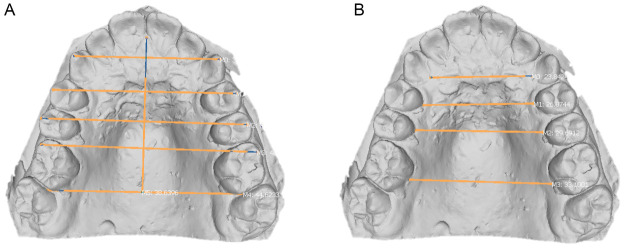
**(A,B)** Measurements performed in subjects with class I malocclusion at the dental level (a) and at the gingival margin level.

**Figure 2:**
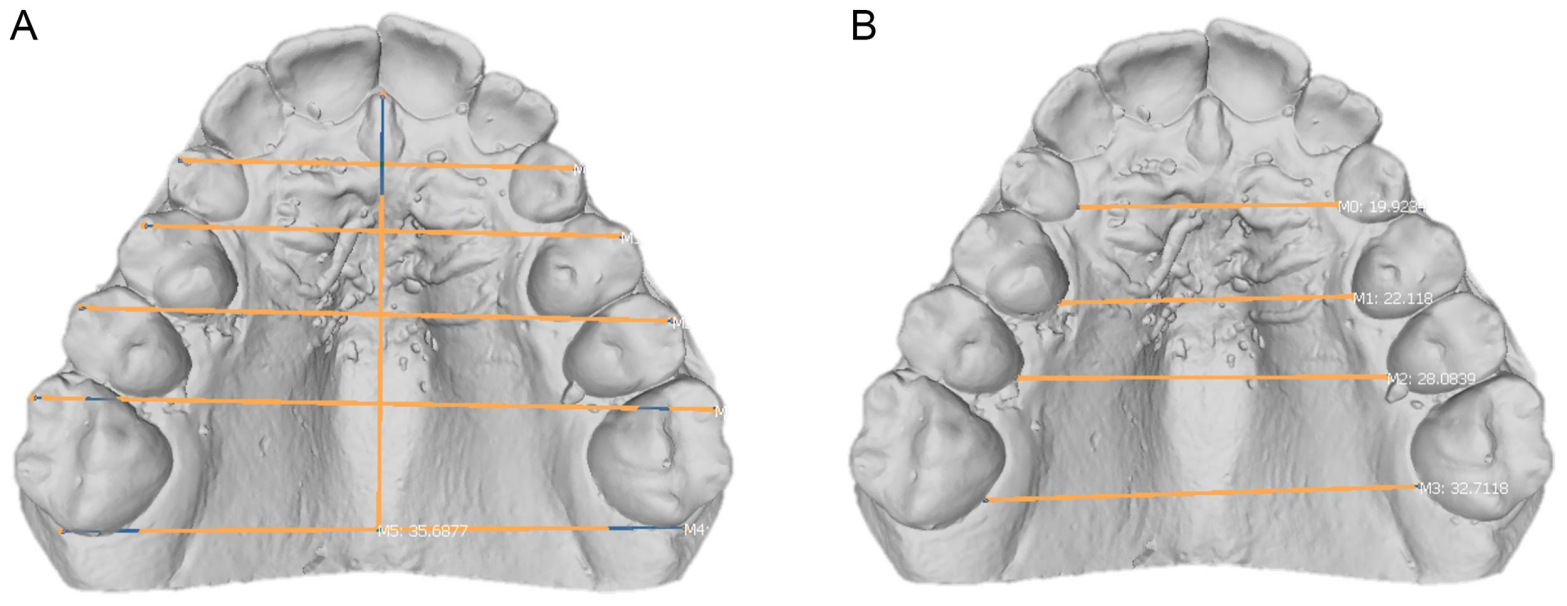
**(A,B)** Measurements performed in subjects Class II Division 1 malocclusion at the dental level (a) and at the gingival margin level.

**Figure 3:**
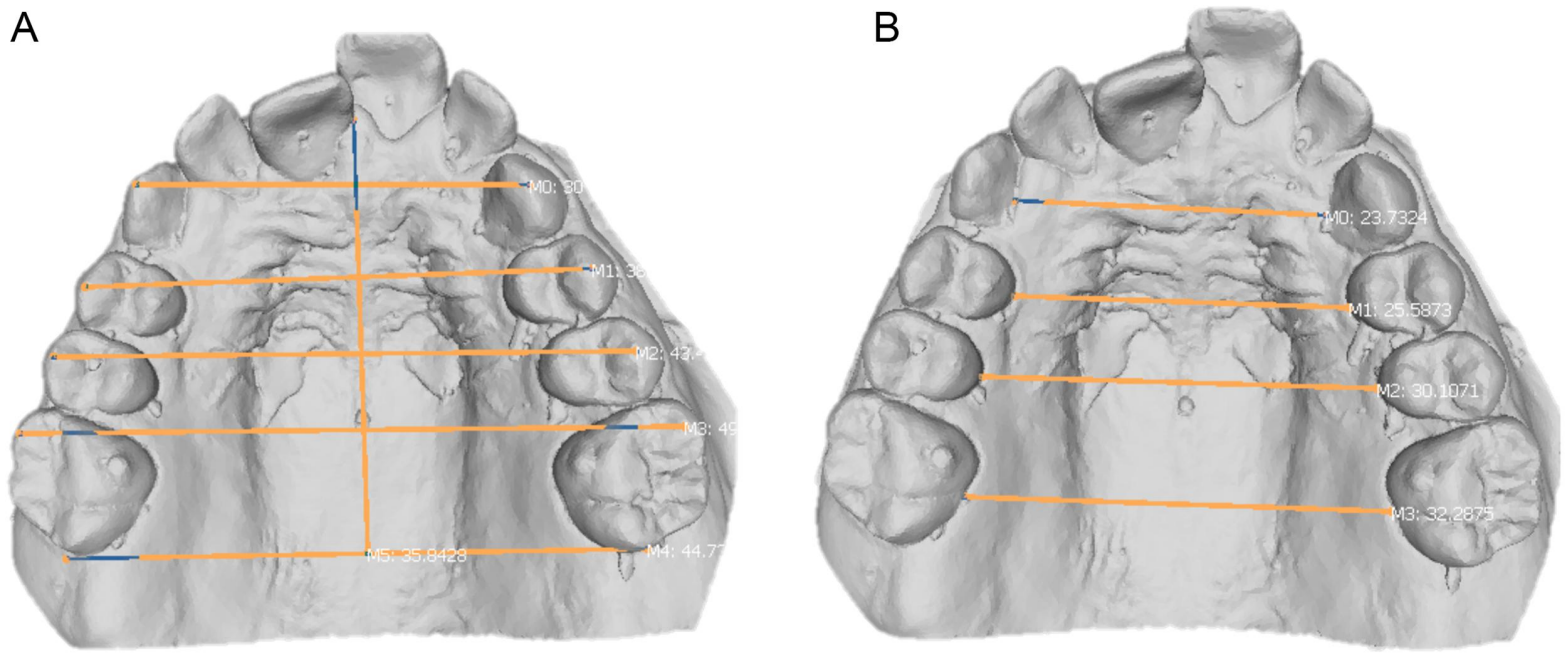
**(A,B)** Measurements performed in subjects Class II Division 2 malocclusion at the dental level (a) and at the gingival margin level.

The plaster models were scanned using an extraoral scanner with a declared accuracy of 20 μm (Dental Wings) and an intraoral scanner (Sirona CEREC). All models were exported in Standard Tessellation Language format (digital STL files). Despite employing two distinct scanning systems, both scanners are commonly utilized in orthodontic digital workflows and yield high-resolution STL models appropriate for linear measurements. The distribution of models across the two scanning systems was comparable among the three malocclusion groups. All digital models were examined with the same software and standardized measurement protocol to reduce possible inter-scanner variability. Transverse measurements of the digitized maxillary arches were performed using MeshLab software. On each maxillary arch scan, six measurements were performed and divided into two groups (Figures 1–3):

(a) **At the level of the dental crown** (canine cusp tips, buccal cusps of the first premolar, second premolar, and mesiobuccal cusp of the first molar)
(b) **At the level of the palatal gingival margin of the dental elements**
  - Distance between gingival margins at the canine level
  - Distance between gingival margins at the first premolar level
  - Distance between gingival margins at the second premolar level
  - Distance between gingival margins at the first molar level
  - Arch length (line passing from the incisive papilla perpendicular to a tangent passing distally to the first molars)

Palatal gingival-margin measurements were considered complementary measurements of maxillary arch morphology and were not intended to provide decompensated transverse dimensions.

In subjects in the mixed dentition stage, when permanent premolars were not fully erupted, the corresponding deciduous molars were used as homologous landmarks for transverse measurements. Therefore, the transverse categories involving the premolar region may include measurements taken on either deciduous or permanent teeth, depending on the individual dentition stage. This approach was adopted to allow comparison of maxillary arch dimensions across the full growing sample, but it may introduce heterogeneity related to dentition stage.

### 2.4 MeshLab software

MeshLab is an open-source software used for processing, visualization, and measurement of 3D digital models. It provides tools for visualization, inspection, and linear measurements on STL files and is widely used for digital model management and analysis in biomedical and technical fields. All anatomical landmarks were manually identified by a trained operator utilizing standardized anatomical reference points on the digital models. To enhance consistency across models with varying morphologies, the same landmark identification protocol and orientation criteria were utilized for all scans prior to measurement collection.

### 2.5 Method error

All measurements were conducted by one calibrated operator. Before the study commenced, a preliminary calibration phase was conducted to standardize the identification of landmarks and the procedures for measurement. In this stage, the operator received training to recognize anatomical reference points based on established criteria using a selection of digital models excluded from the final sample. Furthermore, all STL models were aligned employing identical visualization settings and reference planes in MeshLab to guarantee uniformity of landmark placement and linear measurements across models with varying morphologies. To evaluate intra-operator consistency and method error, 15 randomly chosen models were reexamined four weeks after the initial readings by the same operator. Intra-operator reliability was assessed using the intraclass correlation coefficient (ICC), with values >0.90 for all measurements, excellent intra-operator repeatability.

### 2.6 Statistical analysis

Statistical analysis was conducted utilizing IBM SPSS Statistics version 20 (IBM Corp., Armonk, NY, USA). Descriptive statistics were reported as means and standard deviations (SD). Intergroup comparisons were performed using one-way ANOVA followed by Tukey *post hoc* tests. Statistical significance was set at *p* < 0.05. Cohen's d was calculated to assess effect size and clinical relevance. No *post hoc* power analysis was reported because the study was based on a retrospective convenience sample, as described above. Tukey *post hoc* tests were used to control for pairwise comparisons within each outcome. No global correction was applied across the full set of outcomes; therefore, *p* values close to 0.05 were interpreted with caution.

### 2.7 Ethical considerations

The study was conducted in compliance with the ethical principles outlined in the Declaration of Helsinki. All clinical data were collected and processed in accordance with applicable ethical and privacy requirements. Approval of the research protocol was obtained from the Ethics Committee Protocol No. 2379/CEL.

## 3 Results

Transverse and sagittal maxillary arch dimensions were compared among Class II Division 1 (Class II-Div 1), Class II Division 2 (Class II-Div 2), and Class I (Class I).

Table 1 summarizes the demographic characteristics of the sample, including age and sex distribution across groups.

**Table 1:** Age and sex distribution in the different malocclusion groups.

Group	Females (F)	Males (M)	Mean age ± SD (Years)	Minimum	Maximum
Class I	13	7	10.6 ± 1.5	8.1	13.0
Class II Division 1	8	12	10.8 ± 1.3	7.9	12.5
Class II Division 2	11	9	10.2 ± 1.5	7.8	12.7
Total sample	32	28	10.5 ± 1.4	7.8	13.0

Demographic characteristics of the study sample.

MAX indicates the maximum value; MIN indicates the minimum values; SD indicates standard deviation.

Table 2 summarizes the transverse measurements at the dental level. Individuals with Class II Division 2 malocclusion exhibited notably smaller transverse dimensions than those with Class I malocclusion, especially at the intercanine (3–3 DC), second premolar (5–5 DC), and intermolar (6–6 DC) levels. Individuals with Class II Division 1 malocclusion also showed reduced transverse diameters; however, these differences did not reach statistically significant differences in dental-level measurements when compared to Class I subjects. No statistically significant differences in dental-level transverse dimensions were found between the Class II Division 1 and Class II Division 2 groups.

**Table 2:** Transverse maxillary arch dimensions, pairwise comparisons, and effect sizes.

Measurement	Type	Class II-1 Mean ± SD	Class II-2 Mean ± SD	Class I Mean ± SD	p II-1 vs. I	p II-2 vs. I	p II-1 vs. II-2	d II-1 vs. I (95% CI)	d II-2 vs. I (95% CI)	d II-1 vs. II-2 (95% CI)
3–3	DC	32.3 ± 2.4	31.0 ± 1.8	33.2 ± 2.3	0.336	0.006	0.181	−0.42 (−1.04 to 0.21)	−1.07 (−1.74 to −0.41)	0.59 (−0.05 to 1.22)
4–4	DC	39.4 ± 2.9	38.1 ± 1.9	40.1 ± 2.3	0.645	0.033	0.222	−0.26 (−0.88 to 0.36)	−0.92 (−1.58 to −0.27)	0.52 (−0.11 to 1.15)
5–5	DC	44.4 ± 2.6	42.9 ± 2.1	45.7 ± 3.0	0.272	0.003	0.159	−0.46 (−1.09 to 0.17)	−1.08 (−1.74 to −0.41)	0.64 (0.01 to 1.28)
6–6	DC	49.9 ± 2.4	48.7 ± 2.2	51.6 ± 3.2	0.111	0.004	0.375	−0.60 (−1.24 to 0.03)	−1.03 (−1.70 to −0.37)	0.48 (−0.14 to 1.11)
3–3	DG	23.9 ± 1.6	23.8 ± 1.4	25.5 ± 2.3	0.015	0.009	0.985	−0.84 (−1.49 to −0.19)	−0.92 (−1.57 to −0.26)	0.06 (−0.56 to 0.68)
4–4	DG	26.3 ± 2.4	25.9 ± 2.1	27.1 ± 2.4	0.564	0.251	0.831	−0.31 (−0.94 to 0.31)	−0.52 (−1.15 to 0.11)	0.19 (−0.43 to 0.81)
5–5	DG	30.0 ± 2.0	29.4 ± 1.7	31.3 ± 2.9	0.186	0.027	0.659	−0.51 (−1.14 to 0.12)	−0.80 (−1.45 to −0.16)	0.33 (−0.29 to 0.96)
6–6	DG	33.2 ± 2.1	32.2 ± 2.2	34.8 ± 2.9	0.095	0.003	0.407	−0.64 (−1.28 to −0.00)	−1.01 (−1.67 to −0.35)	0.46 (−0.16 to 1.09)

DC, dental-crown distance; DG, palatal gingival-margin distance; SD, standard deviation; CI, confidence interval. Pairwise *p* values were obtained using Tukey HSD tests. Negative Cohen's d values indicate that the first-mentioned group had a smaller mean.

Measurements taken at the palatal gingival margin level showed notably decreased transverse dimensions in Class II Division 2 individuals compared to Class I individuals at the intercanine (3–3 DG), second premolar (5–5 DG), and intermolar (6–6 DG) levels. Subjects in Class II Division 1 demonstrated a statistically significant decrease only at the intercanine gingival level (3–3 DG) in contrast to Class I subjects. No statistically significant differences were found between the two Class II groups for transverse measurements at the gingival level (Table 2). The largest between-group differences in maxillary transverse measurements were observed between the Class II Division 2 and Class I groups. Comparisons between Class II Division 1 and Class I individuals typically exhibited small-to-moderate effect sizes. Table 3 presents comparisons of sagittal arch lengths. Class II Division 2 subjects showed significantly shorter maxillary arch length than both Class I subjects (*p* = 0.035) and Class II Division 1 subjects (*p* = 0.032).

**Table 3:** Descriptive statistics and intergroup comparisons of maxillary arch length.

Group	MAX	MIN	Mean ± SD	p vs. Class I	d vs. Class I (95% CI)	p vs. Class II-1	d vs. Class II-1 (95% CI)
Class I	47.3	34.3	38.3 ± 3.2	—	—	0.999	−0.01 (−0.63 to 0.61)
Class II Division 1	44.0	31.4	38.4 ± 3.1	0.999	0.01 (−0.61 to 0.63)	—	—
Class II Division 2	39.4	31.5	36.0 ± 2.2	0.035	−0.84 (−1.49 to −0.19)	0.032	−0.88 (−1.53 to −0.23)

MAX, maximum; MIN, minimum; SD, standard deviation; CI, confidence interval. Pairwise *p* values were obtained using Tukey HSD tests.

In mixed dentition subjects, deciduous molars were used as corresponding landmarks when permanent premolars were not fully erupted. Therefore, premolar-region measurements may include homologous deciduous or permanent teeth according to the individual dentition stage.

## 4 Discussion

This study evaluated the transverse and sagittal dimensions of the maxillary arch using three-dimensional digital models in growing subjects with Class I, Class II Division 1, and Class II Division 2 malocclusions. Few previous studies have analyzed and compared arch shape and dimensions between these different malocclusion types (3, 36–40). A comparison between the groups revealed that subjects with Class II Division 2 malocclusion generally had smaller maxillary transverse dimensions than subjects with Class I malocclusion. These reductions were evident in both types of measurements. Subjects with Class II Division 1 malocclusion showed statistically significant differences compared to the Class I group, primarily in intercanine width. Measurements taken at the level of the dental crowns and the palatal gingival margin were intended to provide complementary descriptions of maxillary arch morphology, using different anatomical landmarks (25, 41–43). Differences between dental-crown and palatal gingival-margin measurements may partly reflect dentoalveolar compensation or tooth inclination; however, this interpretation remains hypothetical because direct measurements of dental inclination were not performed. It is also important to distinguish reduced maxillary arch size from a maxillary transverse discrepancy or a maxillary transverse deficiency (44–48). This study evaluated the maxillary arch independently and did not include corresponding transverse measurements of the mandibular arch. Therefore, the observed differences cannot be interpreted as evidence of a maxillomandibular transverse discrepancy. The diagnosis of transverse discrepancy requires evaluation of the interarch relationship, including corresponding mandibular dimensions. However, reduced maxillary transverse dimensions may represent a clinical finding requiring further diagnostic assessment of the maxillomandibular transverse relationship before any treatment decision is made. A true transverse skeletal assessment would require posteroanterior images or appropriate three-dimensional techniques, such as cone beam computed tomography. Lateral cephalometric radiographs do not provide information on transverse skeletal dimensions (49–53). Performing CBCT examinations solely for the purposes of this study, especially in growing subjects and in the absence of a specific clinical indication, would not have been ethically justifiable due to additional exposure to ionizing radiation. The findings of the present study are partially consistent with previous investigations reporting reduced maxillary transverse dimensions in Class II malocclusion. However, differences among studies may be explained by methodological and sample-related factors (54–61). First, age and dentition stage may influence transverse arch dimensions, particularly in growing subjects, because mixed and permanent dentition stages may show different patterns of dental eruption, arch development, and compensatory tooth inclination. Second, ethnic and population-related characteristics may affect baseline arch dimensions and may limit direct comparison across studies. Third, differences in inclusion criteria, particularly whether Class II groups are defined only by dental relationships or by both dental and skeletal parameters, may influence group composition. Finally, measurement methods differ across studies, with some authors using plaster casts, digital models, dental landmarks, gingival landmarks, posteroanterior radiographs, or CBCT-based measurements (62–65). These methodological differences may explain why some studies report more evident transverse reductions in Class II Division 1 subjects, whereas the present study found more consistent reductions in Class II Division 2 subjects. Regarding sagittal dimensions, the Class II Division 2 group had a significantly shorter maxillary arch length than both the Class I and Class II Division 1 groups (66–69). This finding may be related to the lingual inclination of the central incisors, a characteristic of Class II Division 2 malocclusion. Clinically, the identification of reduced maxillary arch dimensions in growing subjects with Class II Division 2 and Division 1 malocclusion indicates the need for a comprehensive transverse diagnostic evaluation (70–74). This evaluation should include the relationship between the maxillary and mandibular arches, posterior occlusion, dental inclination, the presence of a crossbite, functional mandibular displacements, and skeletal characteristics (75–79).

Early recognition of these characteristics may help identify patients requiring closer monitoring or appropriately planned interceptive treatment. From a clinical perspective, early identification of reduced maxillary transverse dimensions in Class II Division 2 patients may be useful during growth, because it can prompt a more comprehensive diagnostic assessment of the transverse relationship before treatment planning. This may include evaluation of maxillary and mandibular arch coordination, posterior occlusion, dental inclination, functional mandibular shifts, and the presence or absence of posterior crossbite (80–83). When a true transverse discrepancy is confirmed clinically, preventive or interceptive strategies may be considered as part of an individualized treatment plan. However, these findings, taken in isolation, do not support a diagnosis of maxillary transverse deficiency and should not be interpreted as an indication for maxillary expansion. Treatment decisions should be based on clinical examination, maxillomandibular interarch relationships, dental inclination, skeletal findings, and individual patient needs (60, 84).

### 4.1 Study limitations

This study has several limitations. The retrospective convenience sample may have introduced selection bias and reduced the generalizability of the findings.

The limited sample size did not allow for analyses adjusted for sex, age, stage of dentition, vertical facial type, recruitment center, or scanner type. Although normodivergent, hypodivergent, and hyperdivergent subjects were represented in each malocclusion group, vertical facial type was not used as a stratification variable. Therefore, its potential influence on maxillary transverse and sagittal arch dimensions cannot be excluded. Given the unequal sex distribution across groups and the known influence of sex on dental arch dimensions, the reported differences should be interpreted as crude, unadjusted associations rather than adjusted effects.

Furthermore, subjects with mixed and permanent dentition were analyzed together, and deciduous and permanent teeth were combined within the same transverse measurement categories when homologous landmarks were required. This may have introduced heterogeneity related to dentition stage and should be considered when interpreting the transverse measurements. In addition, two different scanning systems were used, without a direct assessment of inter-scanner agreement. Although all STL files were analyzed using the same software and standardized measurement protocol, scanner-related measurement variability cannot be completely excluded. No mandibular measurements were performed; therefore, a transverse maxillomandibular discrepancy could not be assessed. The absence of CBCT scans and direct measurements of dental inclination also prevented the assessment of transverse skeletal dimensions or the obtaining of decompensated transverse measurements. All measurements were performed by a single operator, allowing only intra-operator repeatability to be assessed, not inter-operator agreement. Finally, no global correction for multiple comparisons was applied to the entire set of outcomes. Therefore, results with *p*-values close to 0.05 should be interpreted with caution.

## 5 Conclusions

The analysis of transverse and sagittal maxillary dimensions showed that subjects with Class II malocclusion presented reduced transverse dimensions compared with subjects with Class I malocclusion, particularly when measurements were performed at the palatal gingival margin level. These transverse reductions were more pronounced and consistent in Class II Division 2 subjects, who demonstrated significantly reduced intercanine, interpremolar, and intermolar dimensions compared with Class I subjects.

The comparison of arch lengths showed that Class II Division 2 malocclusion presented a significantly shorter maxillary arch length compared with both Class I and Class II Division 1 subjects.

## Data Availability

The original contributions presented in the study are included in the article/Supplementary Material, further inquiries can be directed to the corresponding authors.

## References

[1] Sassouni V. A classification of skeletal facial types. Am J Orthod. (1969) 55:109–23. 10.1016/0002-9416(69)90122-5 5249177

[2] Halimi A Azeroual MF Abouqal R Zaoui F. [A comparative study of the transverse dimensions of the dental arches between class I dental occlusion and Class II1 and Class II2 malocclusions]. Odontostomatol Trop. (2011) 34:47–52. 22457992

[3] Nie Q Lin J. A comparison of dental arch forms between Class II Division 1 and normal occlusion assessed by Euclidean distance matrix analysis. Am J Orthod Dentofacial Orthop. (2006) 129:528–35. 10.1016/j.ajodo.2005.12.012 16627179

[4] Triviño T Siqueira DF Scanavini MA. A new concept of mandibular dental arch forms with normal occlusion. Am J Orthod Dentofacial Orthop. (2008) 133:10.e15–22. 10.1016/j.ajodo.2007.07.014 18174064

[5] W RE. A study of the facial patterns associated with Class I, Class II, Division 1, and Class II, Division 2 malocclusions. Angle Orthod. (1948) 18:12–5.

[6] Andrews LF. The six keys to normal occlusion. Am J Orthod. (1972) 62:296–309. 10.1016/s0002-9416(72)90268-0 4505873

[7] Corruccini RS Pacciani E. “Orthodontistry” and dental occlusion in Etruscans. Angle Orthod. (1989) 59:61–4. 10.1043/0003-3219(1989)059<0061:OADOIE>2.0.CO;2 2646990 10.1043/0003-3219(1989)059<0061:OADOIE>2.0.CO;2

[8] Frölich FJ. A longitudinal study of untreated Class II type malocclusions. Am J Orthod. (1961) 47:148. 10.1016/0002-9416(61)90210-X

[9] Angle EH. Treatment of Malocclusion of the Teeth and Fractures of the Maxillae: Angle’s System. Philadelphia, PA: S. S. White Dental Mfg. Co. (1900).

[10] Baume LJ. Uniform methods for the epidemiologic assessment of malocclusion. Results obtained with the World Health Organization standard methods (1962 and 1971) in south Pacific populations. Am J Orthod. (1974) 66:251–72. 10.1016/0002-9416(74)90290-5 4528845

[11] Brown T Abbott AH Burgess VB. Longitudinal study of dental arch relationships in Australian aboriginals with reference to alternate intercuspation. Am J Phys Anthropol. (1987) 72:49–57. 10.1002/ajpa.1330720107 3826327

[12] Proffit WR Fields HW Moray LJ. Prevalence of malocclusion and orthodontic treatment need in the United States: estimates from the NHANES III survey. Int J Adult Orthodon Orthognath Surg. (1998) 13:97–106. 9743642

[13] Brunelle JA Bhat M Lipton JA. Prevalence and distribution of selected occlusal characteristics in the US population, 1988-1991. J Dent Res. (1996) 75 Spec No:706–13. 10.1177/002203459607502S10 8594094

[14] Josefsson E Bjerklin K Lindsten R. Malocclusion frequency in Swedish and immigrant adolescents–influence of origin on orthodontic treatment need. Eur J Orthod. (2007) 29:79–87. 10.1093/ejo/cjl054 17290019

[15] Buschang PH Stroud J Alexander RG. Differences in dental arch morphology among adult females with untreated Class I and Class II malocclusion. Eur J Orthod. (1994) 16:47–52. 10.1093/ejo/16.1.47 8181550

[16] Perillo L Masucci C Ferro F Apicella D Baccetti T. Prevalence of orthodontic treatment need in southern Italian schoolchildren. Eur J Orthod. (2010) 32:49–53. 10.1093/ejo/cjp050 19706641

[17] Bilgic F Gelgor IE Celebi AA. Malocclusion prevalence and orthodontic treatment need in central Anatolian adolescents compared to European and other Nations’ adolescents. Dental Press J Orthod. (2015) 20:75–81. 10.1590/2177-6709.20.6.075-081.oar 26691973 PMC4686748

[18] McNamara JA. Components of Class II malocclusion in children 8-10 years of age. Angle Orthod. (1981) 51:177–202. 10.1043/0003-3219(1981)051<0177:COCIMI>2.0.CO;2 7023290 10.1043/0003-3219(1981)051<0177:COCIMI>2.0.CO;2

[19] Moyers RE Riolo ML Guire KE Wainright RL Bookstein FL. Differential diagnosis of Class II malocclusions. Part 1. Facial types associated with Class II malocclusions. Am J Orthod. (1980) 78:477–94. 10.1016/0002-9416(80)90299-7 6933855

[20] Franchi L Baccetti T Stahl F McNamara JA. Thin-plate spline analysis of craniofacial growth in Class I and Class II subjects. Angle Orthod. (2007) 77:595–601. 10.2319/070506-275 17605492

[21] Rothstein TL. Facial morphology and growth from 10 to 14 years of age in children presenting Class II, Division 1 malocclusion: a comparative roentgenographic cephalometric study. Am J Orthod. (1971) 60:619–20. 10.1016/0002-9416(71)90202-8 5288849

[22] Rosenblum RE. Class II malocclusion: mandibular retrusion or maxillary protrusion? Angle Orthod. (1995) 65:49–62. 10.1043/0003-3219(1995)065<0049:CIMMRO>2.0.CO;2 7726463 10.1043/0003-3219(1995)065<0049:CIMMRO>2.0.CO;2

[23] Harris JE Kowalski CJ Walker GF. Discrimination between normal and Class II individuals using Steiner’s analysis. Angle Orthod. (1972) 42:212–20. 10.1043/0003-3219(1972)042<0212:DBNACI>2.0.CO;2 4504538 10.1043/0003-3219(1972)042<0212:DBNACI>2.0.CO;2

[24] Franchi L Baccetti T. Transverse maxillary deficiency in Class II and Class III malocclusions: a cephalometric and morphometric study on postero-anterior films. Orthod Craniofac Res. (2005) 8:21–8. 10.1111/j.1601-6343.2004.00312.x 15667642

[25] Garattini G Crozzoli P Valsasina A. [Role of prolonged sucking in the development of dento-skeletal changes in the face. Review of the literature]. Mondo Ortod. (1990) 15:539–50. 2280788

[26] Farronato M Maspero C Abate A Grippaudo C Connelly ST Tartaglia GM. 3D cephalometry on reduced FOV CBCT: skeletal class assessment through AF-BF on Frankfurt plane-validity and reliability through comparison with 2D measurements. Eur Radiol. (2020) 30:6295–302. 10.1007/s00330-020-06905-7 32382843

[27] Inchingolo AD Ferrara I Viapiano F Netti A Campanelli M Buongiorno S. Rapid maxillary expansion on the adolescent patient: systematic review and case report. Children (Basel). (2022) 9:1046. 10.3390/children9071046 35884030 PMC9317392

[28] Stahl F Baccetti T Franchi L McNamara JA. Longitudinal growth changes in untreated subjects with Class II Division 1 malocclusion. Am J Orthod Dentofacial Orthop. (2008) 134:125–37. 10.1016/j.ajodo.2006.06.028 18617112

[29] Douglass J Tinanoff N. The etiology, prevalence, and sequelae of infraclusion of primary molars. ASDC J Dent Child. (1991) 58:481–3. 1783699

[30] Pinho T Lemos C. Dental repercussions of maxillary lateral incisor agenesis. Eur J Orthod. (2012) 34:698–703. 10.1093/ejo/cjr084 21811006

[31] Inchingolo AD Marinelli G Bassi P Lagioia R Inchingolo F Vinjolli F. Comparison of skeletal and airway changes between subjects with Class II malocclusion treated with functional therapy in the pre-pubertal and pubertal phases. Eur J Paediatr Dent. (2025) 26:255–67. 10.23804/ejpd.2025.2544 41383160

[32] Kisling E Høffding J. Premature loss of primary teeth: part III, drifting patterns for different types of teeth after loss of adjoining teeth. ASDC J Dent Child. (1979) 46:34–8. 283078

[33] Quinzi V Marzo G Saccomanno S. Prevention, early diagnosis and timing in orthodontics. Eur J Paediatr Dent. (2025) 26:49–51. 10.23804/ejpd.2025.S06 41384345

[34] Alarashi M Franchi L Marinelli A Defraia E. Morphometric analysis of the transverse dentoskeletal features of Class II malocclusion in the mixed dentition. Angle Orthod. (2003) 73:21–5. 10.1043/0003-3219(2003)073<0021:MAOTTD>2.0.CO;2 12607851 10.1043/0003-3219(2003)073<0021:MAOTTD>2.0.CO;2

[35] Crincoli V Inchingolo AD Marinelli G Lagioia R Bassi P Ciocia C. Evaluation of the possible correlation between dental occlusion and craniomandibular disorders by means of teethan® electromyography: clinical-observational study on 20 patients. J Clin Med. (2025) 14:5508. 10.3390/jcm14155508 40807129 PMC12347565

[36] Maspero C Galbiati G Giannini L Guenza G Farronato M. Class II Division 1 malocclusions: comparisons between one- and two-step treatment. Eur J Paediatr Dent. (2018) 19:295–9. 10.23804/ejpd.2018.19.04.8 30567446

[37] Lux CJ Conradt C Burden D Komposch G. Dental arch widths and mandibular-maxillary base widths in Class II malocclusions between early mixed and permanent dentitions. Angle Orthod. (2003) 73:674–85. 10.1043/0003-3219(2003)073<0674:DAWAMB>2.0.CO;2 14719732 10.1043/0003-3219(2003)073<0674:DAWAMB>2.0.CO;2

[38] Saccomanno S Saran S De Luca M Fioretti P Gallusi G. Prevention of malocclusion and the importance of early diagnosis in the Italian young population. Eur J Paediatr Dent. (2022) 23:178–82. 10.23804/ejpd.2022.23.03.02 36172913

[39] Paglia L. Interceptive orthodontics: awareness and prevention is the first cure. Eur J Paediatr Dent. (2023) 24:5. 10.23804/ejpd.2023.24.01.01 36853207

[40] Bishara SE Bayati P Jakobsen JR. Longitudinal comparisons of dental arch changes in normal and untreated Class II, Division 1 subjects and their clinical implications. Am J Orthod Dentofacial Orthop. (1996) 110:483–9. 10.1016/s0889-5406(96)70054-9 8922506

[41] Millett DT Cunningham SJ O’Brien KD Benson P Williams A De Oliveira CM. Orthodontic treatment for deep bite and retroclined upper front teeth in children. Cochrane Database Syst Rev. (2006) 18:CD005972. 10.1002/14651858.CD005972.pub2 17054268

[42] Tollaro I Baccetti T Franchi L Tanasescu CD. Role of posterior transverse interarch discrepancy in Class II, division 1 malocclusion during the mixed dentition phase. Am J Orthod Dentofacial Orthop. (1996) 110:417–22. 10.1016/s0889-5406(96)70045-8 8876494

[43] Yu Q Pan X Qian Y Fan L. Sagital position of glenoid fossa in angle Class II malocclusion with mandibular retrusion. Shanghai Kou Qiang Yi Xue. (2009) 18:5–9. 19290418

[44] Elsasser WA Wylie WL. The craniofacial morphology of mandibular retrusion. Am J Phys Anthropol. (1948) 6:461–73. 10.1002/ajpa.1330060415 18122644

[45] Braun S Hnat WP Fender DE Legan HL. The form of the human dental arch. Angle Orthod. (1998) 68:29–36. 10.1043/0003-3219(1998)068<0029:TFOTHD>2.3.CO;2 9503132 10.1043/0003-3219(1998)068<0029:TFOTHD>2.3.CO;2

[46] Haynes S. The prevalence of malocclusion in English children aged 11–12 years. In: Report of the Congress. (London: European Orthodontic Society) (1970), p. 89–98. 5287526

[47] Dipalma G Marinelli G Bassi P Lagioia R Calò F Cavino M. Impact of functional therapy on skeletal structures and airways in patients with Class II malocclusion: comparison of treatment in prepubertal and pubertal phases. Life (Basel). (2025) 15:1144. 10.3390/life15071144 40724646 PMC12300978

[48] Gelb M Montrose J Paglia L Saccomanno S Quinzi V Marzo G. Myofunctional therapy part 2: prevention of dentofacial disorders. Eur J Paediatr Dent. (2021) 22:163–7. 10.23804/ejpd.2021.22.02.15 34238010

[49] Inchingolo AD Inchingolo AM Campanelli M Carpentiere V De Ruvo E Ferrante L. Orthodontic treatment in patients with atypical swallowing and malocclusion: a systematic review. J Clin Pediatr Dent. (2024) 48:14–26. 10.22514/jocpd.2024.100 39275817

[50] Cozzani M Mazzotta L Cozzani P. Early interceptive treatment in the primary dentition - a case report. J Orthod. (2013) 40:345–51; quiz 353. 10.1179/1465313313Y.0000000068 24297966

[51] Paglia L. Children’s rights and dental care treatment. Eur J Paediatr Dent. (2024) 25:3. 10.23804/ejpd.2024.25.01.01 38426296

[52] Maspero C Galbiati G Giannini L Farronato G. Sagittal and vertical effects of transverse sagittal maxillary expander (TSME) in three different malocclusion groups. Prog Orthod. (2015) 16:6. 10.1186/s40510-015-0075-z 25907431 PMC4409608

[53] Quinzi V Saccomanno S Manenti RJ Giancaspro S Coceani Paskay L Marzo G. Efficacy of rapid maxillary expansion with or without previous adenotonsillectomy for pediatric obstructive sleep apnea syndrome based on polysomnographic data: a systematic review and meta-analysis. Appl Sci. (2020) 10:6485. 10.3390/app10186485

[54] Ciavarella D Monsurrò A Padricelli G Battista G Laino L Perillo L. Unilateral posterior crossbite in adolescents: surface electromyographic evaluation. Eur J Paediatr Dent. (2012) 13:25–8. 22455524

[55] Quinzi V Federici Canova F Rizzo FA Marzo G Rosa M Primozic J. Factors related to maxillary expander loss due to anchoring deciduous molars exfoliation during treatment in the mixed dentition phase. Eur J Orthod. (2021) 43:332–7. 10.1093/ejo/cjaa061 33215659

[56] Coloccia G Inchingolo AD Inchingolo AM Malcangi G Montenegro V Patano A. Effectiveness of dental and maxillary transverse changes in tooth-borne, bone-borne, and hybrid palatal expansion through cone-beam tomography: a systematic review of the literature. Medicina (Kaunas). (2021) 57:288. 10.3390/medicina57030288 33808680 PMC8003431

[57] Cazzolla AP Campisi G Lacaita GM Cuccia MA Ripa A Testa NF. Changes in pharyngeal aerobic microflora in oral breathers after palatal rapid expansion. BMC Oral Health. (2006) 6:2. 10.1186/1472-6831-6-2 16426457 PMC1388214

[58] Farronato G Giannini L Galbiati G Maspero C. Comparison of the dental and skeletal effects of two different rapid palatal expansion appliances for the correction of the maxillary asymmetric transverse discrepancies. Minerva Stomatol. (2012) 61:45–55. 22402295

[59] Grassia V D’Apuzzo F Ferrulli VE Matarese G Femiano F Perillo L. Dento-skeletal effects of mixed palatal expansion evaluated by postero-anterior cephalometric analysis. Eur J Paediatr Dent. (2014) 15:59–62. 24745595

[60] Inchingolo AM Patano A De Santis M Del Vecchio G Ferrante L Morolla R. Comparison of different types of palatal expanders: scoping review. Children (Basel). (2023) 10:1258. 10.3390/children10071258 37508755 PMC10378123

[61] Maspero C Cavagnetto D Abate A Cressoni P Farronato M. Effects on the facial growth of rapid palatal expansion in growing patients affected by juvenile idiopathic arthritis with monolateral involvement of the temporomandibular joints: a case-control study on posteroanterior and lateral cephalograms. J Clin Med. (2020) 9:1159. 10.3390/jcm9041159 32325675 PMC7230922

[62] Altemus LA. Horizontal and vertical dentofacial relationships in normal and Class II Division I malocclusion in girls 11–15 years*. Angle Orthod. (1955) 25:120–37. 10.1043/0003-3219(1955)025<0120:HAVDRI>2.0.CO;2

[63] Dipalma G Marinelli G Inchingolo F Longo M Di Giulio Cesare M Di Serio S. Clinical efficacy of clear aligners in Class II malocclusion: from pediatric to adult cases-a narrative review. J Funct Biomater. (2025) 16:354. 10.3390/jfb16090354 41003424 PMC12470377

[64] Feres MFN Raza H Alhadlaq A El-Bialy T. Rapid maxillary expansion effects in Class II malocclusion: a systematic review. Angle Orthod. (2015) 85:1070–9. 10.2319/102514-768.1 26516713 PMC8612060

[65] Frilund E Sonesson M Magnusson A. Patient compliance with twin block appliance during treatment of Class II malocclusion: a randomized controlled trial on two check-up prescriptions. Eur J Orthod. (2023) 45:142–9. 10.1093/ejo/cjac046 35968672 PMC10065135

[66] Kalha AS. Early orthodontic treatment reduced incisal trauma in children with Class II malocclusions. Evid Based Dent. (2014) 15:18–20. 10.1038/sj.ebd.6400986 24763171

[67] Khoja A Fida M Shaikh A. Cephalometric evaluation of the effects of the twin block appliance in subjects with Class II, Division 1 malocclusion amongst different cervical vertebral maturation stages. Dental Press J Orthod. (2016) 21:73–84. 10.1590/2177-6709.21.3.073-084.oar 27409656 PMC4944732

[68] McNamara JA Peterson JE Alexander RG. Three-dimensional diagnosis and management of Class II malocclusion in the mixed dentition. Semin Orthod. (1996) 2:114–37. 10.1016/s1073-8746(96)80048-x 9161275

[69] Patano A Inchingolo AM Cardarelli F Inchingolo AD Viapiano F Giotta M. Effects of elastodontic appliance on the pharyngeal airway space in Class II malocclusion. J Clin Med. (2023) 12:4280. 10.3390/jcm12134280 37445315 PMC10342366

[70] Taloumtzi M Padashi-Fard M Pandis N Fleming PS. Skeletal growth in Class II malocclusion from childhood to adolescence: does the profile straighten? Prog Orthod. (2020) 21:13. 10.1186/s40510-020-00313-9 32419086 PMC7231809

[71] Tentolouri E Antonarakis GS Georgiakaki I Kiliaridis S. Masseter muscle thickness and treatment outcomes in children with Class II Division 1 malocclusion. Eur J Paediatr Dent. (2021) 22:298–302. 10.23804/ejpd.2021.22.04.7 35034461

[72] Thiruvenkatachari B Harrison JE Worthington HV O’Brien KD. Orthodontic treatment for prominent upper front teeth (Class II malocclusion) in children. Cochrane Database Syst Rev. (2013) 11:CD003452. 10.1002/14651858.CD003452.pub3 24226169

[73] Varrela J. Early developmental traits in Class II malocclusion. Acta Odontol Scand. (1998) 56:375–7. 10.1080/000163598428356 10066120

[74] Veitz-Keenan A Liu N. One phase or two phase orthodontic treatment for Class II Division 1 malocclusion? Evid Based Dent. (2019) 20:72–3. 10.1038/s41432-019-0049-y 31562403

[75] Inchingolo AM Inchingolo AD Carpentiere V Del Vecchio G Ferrante L Di Noia A. Predictability of dental distalization with clear aligners: a systematic review. Bioengineering (Basel). (2023) 10:1390. 10.3390/bioengineering10121390 38135981 PMC10740623

[76] Zheng J Zhang Y Wu Q Xiao H Li F. Three-dimensional spatial analysis of the temporomandibular joint in adult patients with Class II Division 2 malocclusion before and after orthodontic treatment: a retrospective study. BMC Oral Health. (2023) 23:477. 10.1186/s12903-023-03210-9 37438801 PMC10339615

[77] Zreaqat M Hassan R Samsudin R Stas Y Hanoun A. Three-dimensional analysis of upper airways in Class II malocclusion children with obstructive sleep apnea. J World Fed Orthod. (2022) 11:156–63. 10.1016/j.ejwf.2022.08.001 36155001

[78] Alouini O Rollet D. [Morphological and functional peri-oral changes during early treatment of Class II Division 1 malocclusions using EF line (®) functional education devices]. Orthod Fr. (2018) 89:289–306. 10.1051/orthodfr/2018025 30255844

[79] De Bittencourt Neto AC Saga AY Pacheco AAR Tanaka O. Therapeutic approach to Class II, Division 1 malocclusion with maxillary functional orthopedics. Dental Press J Orthod. (2015) 20:99–125. 10.1590/2176-9451.20.4.099-125.sar 26352852 PMC4594316

[80] Inchingolo AM Inchingolo AD Trilli I Ferrante L Di Noia A De Ruvo E. Orthopedic devices for skeletal Class III malocclusion treatment in growing patients: a comparative effectiveness systematic review. J Clin Med. (2024) 13:7141. 10.3390/jcm13237141 39685600 PMC11642149

[81] Souki BQ Bastos BDC Araujo LFF Moyses-Braga WF Pantuzo MG Cheib PL. Effective and efficient herbst appliance therapy for skeletal Class II malocclusion patient with a low degree of collaboration with the orthodontic treatment. Case Rep Dent. (2015, ) 2015:986597. 10.1155/2015/986597 25861486 PMC4377456

[82] Tanaka OM Fornazari IA Parra AXG De Castilhos BB Franco A. Complete maxillary crossbite correction with a rapid palatal expansion in mixed dentition followed by a corrective orthodontic treatment. Case Rep Dent. (2016) 2016:8306397. 10.1155/2016/8306397 27239351 PMC4864566

[83] Meme’ L Bambini F Dipalma G Sampalmieri F Laforgia A Inchingolo AD. The key role of the palatal expander in orthodontics. *Eur. J. Musculoskelet. Dis.* (2024) 13(3 Suppl 2):S175–82.

[84] Memè L Bambini F Sampalmieri F Pezzolla C Trilli I Sabatelli F. Comparison of palatal expanders and their efficacy: a narrative review. Oral Implantol (Rome). (2024) 16:441–60. 10.11138/oi163.1suppl441-460

